# Ontogenetic and Pathogenetic Views on Somatic Chromosomal Mosaicism

**DOI:** 10.3390/genes10050379

**Published:** 2019-05-19

**Authors:** Ivan Y. Iourov, Svetlana G. Vorsanova, Yuri B. Yurov, Sergei I. Kutsev

**Affiliations:** 1 Yurov’s Laboratory of Molecular Genetics and Cytogenomics of the Brain, Mental Health Research Center, 117152 Moscow, Russia; svorsanova@mail.ru (S.G.V.); y_yurov@yahoo.com (Y.B.Y.); 2 Laboratory of Molecular Cytogenetics of Neuropsychiatric Diseases, Veltischev Research and Clinical Institute for Pediatrics of the Pirogov Russian National Research Medical University, 125412 Moscow, Russia; 3 Research Centre for Medical Genetics, 115522 Moscow, Russia; kutsev@mail.ru; 4 Molecular & Cell Genetics Department, Pirogov Russian National Research Medical University, 117997 Moscow, Russia

**Keywords:** chromosome, chromosome heterogeneity, genomic variations, somatic mosaicism, chromosomal instability, genome instability, aneuploidy

## Abstract

Intercellular karyotypic variability has been a focus of genetic research for more than 50 years. It has been repeatedly shown that chromosome heterogeneity manifesting as chromosomal mosaicism is associated with a variety of human diseases. Due to the ability of changing dynamically throughout the ontogeny, chromosomal mosaicism may mediate genome/chromosome instability and intercellular diversity in health and disease in a bottleneck fashion. However, the ubiquity of negligibly small populations of cells with abnormal karyotypes results in difficulties of the interpretation and detection, which may be nonetheless solved by post-genomic cytogenomic technologies. In the post-genomic era, it has become possible to uncover molecular and cellular pathways to genome/chromosome instability (chromosomal mosaicism or heterogeneity) using advanced whole-genome scanning technologies and bioinformatic tools. Furthermore, the opportunities to determine the effect of chromosomal abnormalities on the cellular phenotype seem to be useful for uncovering the intrinsic consequences of chromosomal mosaicism. Accordingly, a post-genomic review of chromosomal mosaicism in the ontogenetic and pathogenetic contexts appears to be required. Here, we review chromosomal mosaicism in its widest sense and discuss further directions of cyto(post)genomic research dedicated to chromosomal heterogeneity.

## 1. Introduction

The serendipitous establishment of the human karyotype by Tijo and Levan launched the era of cytogenetics in 1956. Until recently, cytogenetic analysis remained probably the most used genetic technique, which had provided voluminous data on chromosomal abnormalities and benign cytogenetic variants [1,2]. As a result, numerous genetic conditions characterized by the presence of cells differing with respect to the chromosome complements (i.e., somatic chromosomal mosaicism or SCM) were reported. Moreover, the idea that all the cells of the human organism (>10^14^) produced by >10^16^ mitoses might share identical genomes/karyotypes had been compromised, suggesting that all humans could be genetically mosaic [3,4]. Nonetheless, an increased number of cells with abnormal karyotypes had been generally supposed to be causative for a variety of human diseases [5,6,7,8]. Currently, intercellular chromosome variations seem to be one of the commonest types of somatic mosaicism in humans.

SCM has become an intriguing focus of biomedical research aimed at discovering missing heritability in human diseases (especially, the heritability “missed” because of ignoring somatic genome variations or genome instability) [3,9]. Thus, SCM and chromosome instability (CIN) are likely to be either mechanisms or pathogenetic cascade elements of a wide spectrum of diseases and to mediate interindividual genetic diversity, prenatal development, and aging [3,4,8,10,11]. SCM/CIN manifested as aneuploidy has global significance, which requires further analysis of molecular and cellular pathways to the generation and prevention of numerical and structural chromosome aberrations in somatic cells [12]. In this light, one has to address network-/pathway-based (post-genomic) analyses of alterations to genome stability maintenance, cell cycle regulation, mitotic chromosome segregation, and programmed cell death for understanding origins and mechanisms for SCM/CIN.

The investigation of SCM/CIN in human embryos and fetuses is an important part of cytogenomic/cytogenetic research. Apparently, SCM/CIN (i.e., aneuploidy) contributes significantly to normal and pathological prenatal development in humans [12,13]. Considering the effects of aneuploidy on cellular homeostasis [14,15], it is not surprising that SCM and CIN manifesting as numerical chromosome abnormalities are involved in human prenatal mortality and/or natural cellular selection in human embryos and fetuses [5,7,12,13,15]. Similarly, these processes appear to mediate aging [7,10] and cancer [11] at the cellular level. Therefore, the understanding of origins, mechanisms, incidence, and phenotypic consequences of SCM/CIN requires a consideration of them in the ontogenetic context [16]. Hence, studies of molecular and cellular pathways to pathologic conditions mediated by SCM/CIN should consider the dynamic nature of somatic genome variations.

Recent opportunities for uncovering molecular and cellular pathways to somatic mosaicism allow SCM and CIN to be re-considered in the light of cyto(post)genomic research for further studies of chromosomal heterogeneity. Here, we review the commonest types of chromosomal heterogeneity—SCM and CIN—in the ontogenetic and pathogenetic contexts in the post-genomic era. We discuss the origins of SCM and CIN and their role in prenatal development and genetic architecture of different tissue types. Further, we address the contribution of SCM/CIN to human diseases. Finally, we consider somatic genome variations at the chromosomal level involved in human aging.

## 2. Chromosomal Mosaicism during Early Development

**Figure 1:**
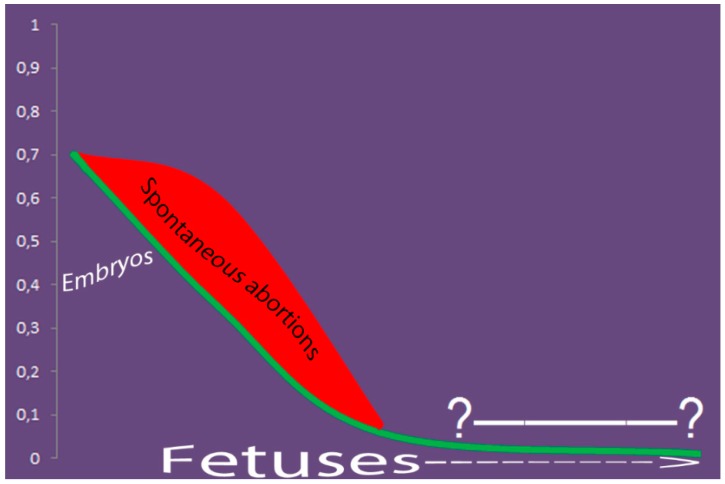
Profiling of decrease trend in somatic chromosomal mosaicism (SCM) rates throughout the prenatal development (according to [3,13,16,18,19,22,23,24,25,28,31,35]). Up to 70% of preimplantation embryos exhibit SCM/chromosome instability (CIN). About 25% of viable fetuses exhibit SCM/CIN (including chromosomal mosaicism confined to embryonic and extraembryonic tissues); in addition, up to 25% non-viable fetuses (spontaneous abortions) exhibit SCM at this development stage, as well. Later gestational periods are poorly addressed in terms of SCM. Nonetheless, cytogenetic analyses in the second and third trimester (prenatal diagnosis) show a significant decrease of SCM incidence as to cytogenetic studies of spontaneous and induced abortions. Therefore, one can suggest the existence of selective pressure against fetuses with SCM/CIN in early human ontogeny. The x-axis corresponds to the timeline from conception to birth, whereas the y-axis corresponds to the proportion of embryos/fetuses affected by SCM/CIN; the question marks fetal periods, which are poorly addressed in terms of SCM/CIN by molecular cytogenetic techniques.

At least 10% of human pregnancies are supposed to be affected by aneuploidy [15,17]. The early human prenatal development is hallmarked by chromosomal mosaicism and CIN. Actually, about 70% (or even 90%) of embryos are suggested to be mosaic or to be affected by CIN [18,19,20]. Here, it is important to indicate that single-cell pathologic events leading to SCM/CIN in early embryos are likely to be more detectable as to other embryonic/fetal and extraembrionic samples, inasmuch as the cellular populations are small. Nonetheless, the early human prenatal development seems to be associated with increased somatic mutation rates [21]. Further stages of prenatal development are characterized by a decrease in proportion of embryos affected by SCM/CIN and/or non-mosaic chromosomal abnormalities, which seems to correlate with the formation of primary germ layers [16,22,23]. Simultaneously, developmental tissue-specific mosaicism and CIN occur. Thus, embryonic/fetal tissues (fetal brain) exhibit CIN manifesting as aneuploidy in 30–35% of cells. Additionally, nearly one-quarter of fetuses demonstrate brain-specific SCM [24,25]. Alternatively, mosaicism confined to placenta is observed in a small but significant proportion of pregnancies (0.5–1%) [26,27]. The impact of numerical chromosome abnormalities on cellular/fetal homeostasis lies at the origins of an appreciable proportion of spontaneous abortions (fetal deaths). In the light of SCM, up to 25% of spontaneously aborted fetuses are chromosomally mosaic [28,29,30]. Although the intrinsic effect of CIN on fetal homeostasis remains to be assessed, the impact of low-level mosaicism on fetal/embryonic viability seems to be elusive [31,32]. During prenatal diagnosis, the incidence of SMC still remains appreciable [33,34]. However, since systematic evaluations of SMC/CIN in prenatal diagnosis have been generally made using karyotyping, the real incidence is hardly evaluable. In this context, one should bear in mind that cytogenetic (molecular cytogenetic) prenatal diagnosis is usually performed in pregnancies with an increased risk of chromosomal abnormalities [33]. In newborns, low-level SCM may be detected in almost all individuals [3,35]. This phenomenon is likely to be a trace of a decrease in rates of SCM/CIN due to natural cellular selection [3,7,12,25]. Alternatively, due to the devastative effect on cellular homeostasis, SCM/CIN might result in mosaic chromosomal pathology with recognized patterns of malformations after birth [14,36]. The latter is relatively uncommon in newborns studied by karyotyping [37,38]. However, the application of single-cell molecular cytogenetic techniques gives an impression that SCM and CIN rates are significantly higher than the rates revealed by cytogenetic analyses [16,35,39]. Unfortunately, SCM and CIN are poorly studied by molecular cytogenetic techniques in unselected population at perinatal period. However, a trend of a decrease in the rate of interindividual genetic variation during prenatal development due to natural selection appears to exist. Figure 1 depicts this trend with an attempt to highlight spontaneously aborted fetuses with SCM as the most probable cause for the decrease.

In clinical populations, SCM is repeatedly reported [7,39]. Thus, mosaic chromosome aberrations are associated with a variety of abnormal phenotypes [40]. A wide spectrum of brain diseases (i.e., neuropsychiatric and neurodevelopmental disorders) are associated with somatic mosaicism including SCM and CIN [39,41,42,43,44,45]. For instance, SCM seems to be the commonest type of genetic changes in autistic individuals [46]. Consequently, it is to conclude that SCM does have an impact on the phenotype.

**Figure 2:**
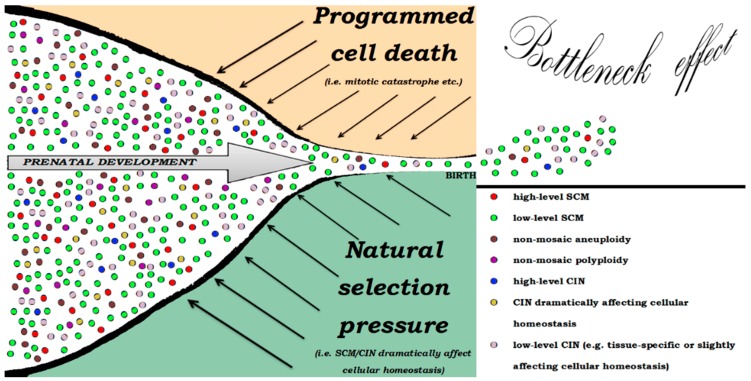
Schematic representation of the bottleneck model for the explanation of selective pressure in fetuses with non-mosaic chromosomal aberrations (mainly, aneuploidy, and polyploidy), SCM and CIN throughout prenatal development. The bottleneck effect may be achieved both by the natural selection pressure and by programmed cell death.

Paradoxically, SCM and CIN may serve either or both as a mechanism for regulation of cell numbers and as a cause for prenatal mortality (postnatal morbidity). The former is likely to be an integrated part of human development, whereas the latter is likely to the result of alterations to the former. It appears that SMC and CIN are elements of a global developmental system targeted at endogenous cellular selection through programmed cell death (i.e., mitotic catastrophe) in addition to the natural selection pressure achieved through dramatic impact of mosacism and genomic instability on cellular/fetal homeostasis [12,16,47,48,49,50]. Accordingly, we propose a bottleneck model for the description of SCM and CIN effects on human prenatal development (Figure 2). The bottleneck effect might be a mechanism for the ontogenetic changes in the rates of SCM and CIN.

## 3. Tissue-Specific Chromosomal Mosaicism

Addressing chromosomal imbalances in primary germ layers and their derivatives suggests the existence of tissue-specific SCM. The level and location of mosaicism depends on the stage of development of the organism when the error in division occurs [47,51,52]. Indeed, humans exhibit tissue-specific somatic mosaicism with/without pathological consequences requiring an examination of different tissues (cell types) when mosaicism is suspected [7,52,53]. However, there has been no consensus about the contribution of tissue-specific SCM/CIN to normal or pathological tissue architecture mainly due to the technological problems of the detection [52,54]. Still, SCM confined to unaffected human tissues does exist [55]. Molecular genetic analyses of human tissues (i.e., bladder, blood, brain, breast, liver, lung, prostate, skin, stomach, and thyroid) evidences for mosaicism rates differing with respect to a tissue [23,53,56]. Brain-specific mosaicism might be of special importance because of the central nervous system organization (each neuron may form up to several thousand connections to other neurons affecting their functioning). Thus, molecular cytogenetic analysis (i.e., FISH(fluorescence in situ hybridization)-based analysis of single neural cells) has shown 0.1–2% aneuploid cells accounted per homologous chromosome (~5–12% in terms of the entire genome) in the unaffected brain (for review, see [55,57,58]). Additionally, there are long-standing evidences for pathological impact of tissue-specific SCM on brain functioning in cases of chromosome abnormalities [7,59,60,61]. Finally, it is important to mention that the abnormal cells are able to affect almost exclusively one cell type/tissue, and that the level of detectable mosaicism depends on the distribution and location of abnormal cells in an organism [7,52,53].

Similar to studies of adverse effects of somatic mosaicism, the results of studying tissue-specific SCM are strongly dependent on technological factors [57,58,61]. For instance, single-cell sequencing analyses have indicated 0.7–2.7% of karyotypically abnormal cells (in terms of the entire genome) in the unaffected human brain [62,63,64]. The discrepancy of the results might be explained by the advantages/disadvantages of molecular cytogenetic and single-cell sequencing techniques. FISH -based approaches to single-cell analysis of interphase chromosomes have extremely high cell scoring potential (>10,000 cells per probe/analysis), but lack a possibility of efficient analysis of all chromosomes (chromosomal regions) in a single cell/nucleus [65,66]. Contrariwise, single-cell sequencing has extremely low cell scoring potential (~100 cells), but offers the possibility of whole cellular genome analysis [67,68]. It is to note that studies combining molecular cytogenetic/genetic and bioinformatic approaches appear to provide more relevant single-cell genomic data [25,57,69,70,71]. Thus, data on somatic genomic variations uncovered by FISH-based techniques and by single-cell next-generation sequencing are to be fused for reliable analysis of tissue-specific SCM and CIN.

## 4. Mosaic Chromosome Abnormalities

Theoretically, almost all kinds of chromosome abnormalities might be mosaic [40]. With the introduction of molecular cytogenetic and whole-genome scan techniques, it has been shown that mosaic structural chromosome abnormalities (including submicroscopic copy number variations) are found in at least 1.7–4% of individuals from clinical cohorts [3,6,72,73,74,75,76]. Interestingly, a number of structural chromosome abnormalities (e.g., ring chromosomes and structurally rearranged chromosome Y) are commonly involved in dynamic mosaicism (formation of new chromosomal rearrangements from an already abnormal chromosome), inasmuch as the rearranged chromosomes are instable during cell division [77,78,79,80,81]. These types of mosaicism appear to model pathways to SCM and CIN.

In humans, almost all non-mosaic monosomies and mosaic monosomies affecting significant propositions of cells (usually, >30% of cells) are incompatible with life. An exception is monosomy of chromosome X [3,82,83]. However, it is suggested that all the liveborn cases of the X chromosome monosomy are mosaics (cryptic mosaics) inasmuch as non-mosaic monomsomy of chromosome X (a common finding in early spontaneous abortions) is likely to be incompatible with life, as well [83]. Mosaic aneuploidies are common in humans and are associated with a variety of pathogenic conditions [7,40,84,85].

Trisomy of chromosome 1 is extremely rare due to the size, number of genes, and gene enrichment. There are only several reports on mosaic trisomy of chromosome 1 associated with severe non-specific phenotypes [40,86].

Trisomy of chromosome 2 is detected in ~4% of karyotyped spontaneous abortions with chromosome abnormalities. Confined placental mosaicism for trisomy of chromosome 2 is relatively frequent in human conceptuses [87,88]. In newborns, mosaic trisomy of chromosome 2 is extremely rare and is occasionally associated with hypomelanosis of Ito [89,90].

At least seven cases of mosaic trisomy of chromosome 3 revealed by postnatal cytogenetic diagnosis have been reported. Phenotypically, these cases are variable probably because of differences in mosaicism ratios [91,92].

Trisomy of chromosome 4 is generally associated with spontaneous abortions [84]. However, no fewer than four cases of mosaic trisomy of chromosome 4 in liveborns characterized by phenotypic similarity have been reported [93].

Currently, seven cases of mosaic trisomy of chromosome 5 may be found in the available literature. It is suggested to be one of the most severe forms of mosaic autosomal trisomy [40,94].

Mosaic trisomy of chromosome 6 has not been reported in liveborns. This numerical chromosome abnormality is rarely detected in spontaneous abortions, as well [40,84].

Mosaic trisomy of chromosome 7 (non-mosaic trisomy of chromosome 7 is extremely rare) is repeatedly identified in human fetuses during prenatal diagnosis or cytogenetic evaluation of spontaneous abortions. A large proportion of these cases are associated with confined placental mosaicism [87,88]. To date, about 10 liveborn cases affected by mosaic trisomy of chromosome 7 have been described. Four cases exhibited uniparental disomy of chromosome 7 in addition to trisomy suggesting trisomy rescue as a mechanism for mosaicism formation. These cases have exhibited severe phenotypes. A number of postnatal cases exhibiting mosaic trisomy of chromosome 7 have been associated with hypomelanosis of Ito and Silver-Russell syndrome [95,96,97].

Mosaic trisomy of chromosome 8 is as common as mosaic trisomy of chromosome 7 in human fetuses. It is also frequently associated with confined placental mosaicism [87]. In liveborns, mosaic trisomy of chromosome 8 is much more common than mosaic trisomies involving previously mentioned autosomes. In theory, the incidence is suggested to be 1/25,000–1/50,000 in the general population. More than 120 cases have as yet been reported. Clinically, this condition is designated as trisomy 8 mosaicism syndrome [84,98,99].

Mosaic trisomy of chromosome 9 has been identified in more than 40 cases. Having a recognizable pattern of malformations, this condition is determined as a chromosomal syndrome [40,100].

Mosaic trisomy of chromosome 10 is extremely rare. At least 10 reports about this condition have been published [84,101]. However, it seems that a number of these reports (especially, those assessed without high-resolution banding karyotyping) have misrecognized trisomy of chromosome 8 and have been erroneously considered as trisomy of chromosome 10. This suggestion originates from suspicious phenotypic similarity of these cases and cases of chromosome 8 trisomy [40].

Mosaic trisomy of chromosome 11 appears to be incompatible with prenatal development. This is not surprising as it appears to cause acardia (heart absence) [84,102].

Mosaic trisomy of chromosome 12 has been reported in nine cases with variable phenotypes [103]. Here, it is apposite to mention Pallister-Killian syndrome, which is the result of mosaic tetrasomy 12p, inasmuch as this syndrome is consistently shown to be associated with tissue-specific mosaicism (for more information, see [104,105]).

Trisomy of chromosome 13 causes Patau syndrome, a severe genetic disease associated with multiple congenital malformations and short lifespan. The syndrome affects 1/10,000–1/20,000 liveborns; mosaic trisomy is likely to account for 5% of Patau syndrome cases [106,107,108].

Mosaic trisomy of chromosome 14 has been observed in more than 40 cases. In some of them, uniparental disomy has been detected suggesting the trisomy rescue as a mechanism for mosaicism formation as in cases of mosaic chromosome 7 trisomy [109,110].

Mosaic trisomy of chromosome 15 is extremely rare. No fewer than 10 reports about this chromosomal imbalance in liveborns have been published [111]. As in cases of mosaic trisomy of chromosomes 7 and 14, trisomy rescue is common in prenatal cases of mosaic trisomy of chromosome 15; as a result, uniparental disomy associated with disorders of genomic imprinting (Prader-Willi and Angelman syndromes) may be observed [112].

Trisomy of chromosome 16 is the commonest aneuploidy found in spontaneous abortions [17]. It is relatively frequently involved in confined placental mosaicism [87,88]. Trisomy of chromosome 16 is, however, rarely identified in newborns due to the incompatibility with normal prenatal development. Notwithstanding, there have been reported more than 80 cases of mosaic trisomy of chromosome 16 with variable phenotypes [113,114].

Mosaic trisomy of chromosome 17 has been reported in approximately 12 cases. This trisomy is a picturesque example of tissue-specific mosaicism, being present in skin fibroblasts and absent in blood cells [115,116].

Trisomy of chromosome 18 is the cause of Edwards syndrome, the incidence of which achieves 1/6000. Only 10% of children survive beyond the first year. Approximately 5% of Edwards syndrome cases are suggested to be mosaic. Currently, no fewer than 40 descriptions of cases of mosaic trisomy of chromosome 18 have been reported [117,118,119].

Since chromosome 19 is an extremely gene-rich chromosome, it is not surprising that mosaic trisomy of chromosome 19 has not been reported [40,84]. Still, there is a report on a stillborn male with mosaic trisomy 19 [120], which might be another chromosomal imbalance erroneously identified because of low banding resolution.

Mosaic trisomy of chromosome 20 has been reported in more than 40 liveborn cases. Prenatally, it is usually associated with confined placental mosaicism. Liveborns with mosaic trisomy of chromosome 20 demonstrate variable phenotypes, which have, however, a recognizable pattern of malformations. Associations of mosaic trisomy of chromosome 20 with hypomelanosis of Ito have been reported, as well [87,121,122].

Trisomy of chromosome 21 causes the commonest chromosomal disorder in humans, Down syndrome, which occurs at a frequency of at least 1/700–1/800 live births. The overwhelming majority of individuals with Down syndrome have non-mosaic trisomy of chromosome 21. Currently, it is suggested that 1.3–5% of all people with Down syndrome phenotype (classical and subtle forms) have mosaic trisomy of chromosome 21. In terms of the general population, the incidence of mosaic trisomy of chromosome 21 with phenotypic manifestations resembling Down syndrome is likely to range between 1/16,670 and 1/41,670 conceptuses or liveborns [123,124,125,126,127].

Trisomy of chromosome 22 is generally considered the second most common aneuploidy found in spontaneous abortions, and is frequently confined to placenta [84,87]. Mosaic trisomy of chromosome 22 has been described in more than 20 patients with variable and severe manifestations [128,129]. The incidence at birth has been estimated as 1/30,000–1/50,000. Additionally, there are communications describing non-mosaic trisomy of chromosome 22 in liveborns [130]. It is highly likely that all these patients are cryptic tissue-specific mosaics.

Mosaic sex chromosome aneuploidies are common in humans and have a plethora of phenotypic outcomes in addition to gonosomal (sex chromosome) syndromes (i.e., Turner syndrome, Triple X syndrome, Klinefelter syndrome etc.) [131]. As mentioned before, Turner syndrome, which is associated with a variety of chromosome X imbalances leading to a loss of the critical X chromosome regions (for review, see [132]), is likely to result from cryptic or tissue-specific mosaic monosomy [83]. Mosaic X chromosome monosomy may produce extremely variable phenotypes (i.e., reduced penetrance of Turner syndrome phenotype) [133] allowing to hypothesize that it may mediate complex diseases [3,7]. Mosaic X chromosome loss and other sex chromosome aneuploidies (mainly mosaicism for additional chromosomes X in male and female karyotypes) seem to be involved in the etiology of mental illness [46,73,134,135] and autoimmune diseases [136]. Low level mosaic abnormalities (aneuploidy) of chromosome X have been associated with recurrent miscarriages, as well [137,138]. Mosaic loss of chromosome X has long been associated with aging [139] (see part 7). In general, due to a milder phenotypic effect of sex chromosome aneuploidies as to autosomal aneuploidies, there have been numerous attempts to associate complex diseases with gonosomal mosaicism. Some of these attempts have been successful (for more details, see part 5).

Since polyploidy severely affects organismal homeostasis in humans, gains of additional chromosomal sets are generally accepted to be incompatible with life [40,140,141]. Mosaic or regular polyploidy is a frequent cytogenetic finding in spontaneous abortions [28,29,30,142]. However, one can find communications dedicated to postnatal occurrence of polyploid cells in human tissues (i.e., liver and central nervous system) [143,144]. Molecular cytogenetic and genomic analyses of single cells have evidenced that polyploid cells are extremely rare in human tissues [7,25,52,56,57,58,145]. Nonetheless, it is possible that mosaic polyploidy mediates cell death in postmitotic cells experienced endoreduplication (endomitosis) [146,147].

Mosaic aneuploidy and polyploidy are common cytogenetic findings in spontaneous abortions. These are assumed to be devastative in an appreciable proportion of fetuses [28,48,145]. However, low-level mosaics are likely to survive until birth and present milder phenotypic manifestations of a chromosomal disorder [5,6,7,8,39,40,41,42,43,52,84,86]. Furthermore, SCM and CIN might be a mechanism for complex diseases, especially, if confined to a tissue or specific cell type [3,4,5,6,7,9,10,11,12,39,52,54,57,61,70]. Accordingly, SCM/CIN should be a focus of cytopostgenomic research aimed at uncovering disease mechanisms (disease-causing pathways) in its widest sense.

## 5. Chromosomal Heterogeneity, Somatic Mosaicism and Human Disease

In the post-genomic era, specific attention is paid to chromosomal heterogeneity and, more specifically, SCM and CIN. Such attention is likely to result from new opportunities offered by post-genomic technologies (i.e., whole-genome and systems biology analyses of molecular and cellular pathways mediating the effects of genomic/chromosomal variations on cellular and organismal homeostasis) [49,52,148,149,150,151,152]. Intercellular heterogeneity is likely to be achieved by structural and behavioral variability/instability of the genome at all levels of processing genetic information (genomic, epigenomic, proteomic and metabolomic) [153,154,155]. Additionally, high rates of the instability are generated during storage and transmitting of the genetic information (i.e., DNA damage, cell cycle/mitotic checkpoint errors) [50,52,55,105]. Since chromosome number/structure changes because of cell cycle and DNA replication errors are common, SCM and CIN (genomic instability) might be an abundant source of dynamic intercellular variability of the genome in health and disease [3,52,152].

There appear to be three main routes to SCM and CIN. The first one has already been mentioned and is referred to as trisomy/aneuploidy rescue (extra chromosomes are lost during cell division at the earliest ontogenetic stages), which occurs during prenatal development and may lead to uniparental disomy [95,112]. CIN has repeatedly been shown to be caused by mutations in individual genes implicated in a variety of molecular and cellular pathways, which are required for maintaining genome stability, cell cycle progression, and clearance of genetically abnormal cells via programmed cell death (for more details, see [156,157,158]). However, cellular populations with these mutations are rare in contrast to cases of SCM and CIN exhibiting altered functioning of the aforementioned pathways. Therefore, there should be other types of alterations to these pathways resulting in CIN and/or SCM. It has been previously shown that environmental, epigenetic, and chromosomal alterations to the pathways involved in maintenance of genome stability, cell cycle, and programmed cell death are able to produce genomic instability (for details, see [159,160,161]). For instance, copy number changes affecting genes implicated in the cell cycle pathway are able to mediate SCM [162]. More sophisticated system biology approaches to determine causes and consequences of chromosomal variations have shown that a wide spectrum of microscopic and submicroscopic genomic variations and environmental effects are able to cause CIN/SCM [163,164,165,166,167]. Thus, to obtain an integrated view on CIN and SCM, which includes all exogenous and endogenous elements, a “multidimensional” evaluation of genomic changes at genomic, epigenomic, proteomic and metabolomic levels are required [11,12,161,168]. To get an example of a chromosomal imbalance disrupting molecular pathways to genome instability, one can address our previous case-report on 5p13.3p13.2 duplication, which has been associated with CIN manifested as aneuploidy [169]. Finally, somatic aneuploidy per se may produce instability in transmitting genetic information during the cell cycle and become, thereby, a source for CIN leading to a wide spectrum of pathologic conditions [11,157,170]. Almost all these mechanisms of chromosome heterogeneity have been observed in chromosomal and complex diseases.

In addition to disease-causing chromosome abnormalities (part 4), CIN and SCM have been thoroughly evaluated in cancer and non-cancerous brain diseases. Cancer has long been associated with mosaic aneuploidy or other types of SCM/CIN confined to malignant cell lines. Specificity of CIN appears to determine essential properties of malignancies. The role of aneuploidy and related CIN/SCM types is highly dependent on a variety of factors (i.e., co-occurrence of different CIN types, cellular microenvironment etc.) in contrast to specific chromosomal aberrations (i.e., translocations, deletions, gene amplifications). Nonetheless, CIN/SCM is an integral component of the theories describing how genome variability mediates cancer formation and progression [171]. It is suggested that the origins of cancer-causing SCM/CIN results from failures to repair multitudes of lesions to chromosomal DNA occurring systematically over the lifespan of a cell [172,173]. The diverse molecular and cellular changes leading to these failures seem to be the basis of numerous pathways to cancer [10,152,161]. The diversity causes extended chromosomal heterogeneity [152], which is able to produce extremely variable cellular phenotypes [174]. Interestingly, that cancer can be a result of somatic mosaicism (including SCM) [175], tissue-specific mosaicism [176], and aging-related mosaicim [177]. Moreover, specific SCM and CIN are likely to lie at the origin of cell escaping natural selection pressure, the result of which is malignant cell lines [178]. Finally, the specificity of CIN manifestations (i.e., aneuploidy or structural chromosomal abnormalities) is able to define the way of cancer propagation and the response to therapeutic interventions [179]. The investigation of cancer pathways highlights the mechanisms and consequences of SCM and CIN, which might be relevant to other genetic diseases mediated by these ubiquitous processes [7,70,147,180,181,182]. Consequently, it is important to establish whether there is a difference between malignant and non-malignant SMC/CIN. Fortunately, future post-genomic studies are able to provide such data.

Brain diseases are probably the best studied in the context of somatic mosaicism (SCM) and non-malignant CIN [7,57,58,183,184,185,186]. Studies of SCM and CIN in the normal and diseased brain has been formed an emerging field of molecular neurocytogenetics [7,57]. Somatic mosaicism has been recently recognized as an important cause for neuronal genomes diversification and neuropsychiatric disease [187]. More precisely, neurodevelopmental, neuropsychiatric and neurodegenerative disorders have been repeatedly found to exhibit SCM and/or CIN.

Neurodevelopmental diseases have been associated with different types of somatic mosaicism [39,42,43,45,188]. Autism spectrum disorders exhibit high rates of somatic mosaicism manifesting as chromosomal imbalances and single-nucleotide mutations [46,73,189,190]. Interestingly, autistic males exhibit SCM involving chromosome X, which may occur exclusively in males (extra chromosome X in male karyotypes) [46]. This observation has been further used to suggest SCM as one of the reasons for male preponderance in autistic disorders [191,192]. Additionally, a number of autism cases have demonstrated CIN (aneuploidy and chromothripsis) [41,73,193]. It is highly probable that these intercellular genomic variations are elements of pathogenetic cascade or a kind of pathway to brain dysfunction mediated by SCM/CIN [149,185,194,195]. One can suggest that tissue-specific studies may shed light on the role of SCM/CIN in the pathogenesis of autistic spectrum disorders.

The schizophrenia brain has been among the first targets of studying SCM in the neurocytogenetic context (i.e., analysis of SCM and CIN in the brain to highlight their roles in health and diseases). Initially, SCM/aneuploidy involving chromosomes 18 and X has been found in two schizophrenia cases (postmortem brain samples) [183]. More recently, it has been shown that sex chromosome aneuploidy (sex chromosome-specific CIN) may be significantly increased in the brain of individuals with schizophrenia and comorbid psychiatric disorders [196]. Additionally, it has been shown that SCM involving gain/loss of chromosome 1 and chromosome 1-specific CIN in the diseased brain is able to contribute to schizophrenia pathogenesis in at least of a proportion of cases [197]. It is to note that chromosome 1 aneuploidy is extremely rare in humans. In total, overall levels of mosaic aneuploidy/CIN are three-fold higher in the schizophrenia brain than in controls [198]. The schizophrenia seems to be also associated with brain-specific submicroscopic genomic variations (copy number variations) [199].

SCM/aneuploidy has long been proposed as a possible mechanism for neurodegenerative diseases [7,184,200,201]. Furthermore, DNA damage and DNA reparation deficits have been systematically associated with neurodegeneration [202,203]. CIN has been previously associated with neurodegenerative processes in a number of hereditary diseases (CIN syndromes) [180,182]. Ataxia telangiectasia (an autosomal recessive CIN syndrome characterized by progressive neurodegeneration and cerebellar ataxia, telangiectasia, immunodeficiency, cancer susceptibility, and radiation sensitivity) demonstrates selective cerebellar neurodegeneration and near lack of degenerative processes in non-cerebellar brain areas [204,205]. The mechanism for the neurodegeneration in ataxia telangiectasia is CIN (5–20-fold increase of non-random interphase chromosome breaks and structural/numerical abnormalities affecting chromosomes 14) confined to the cerebellum [180]. It is noteworthy that SCM/CIN manifesting as aneuploidy is likely to be a common mechanism for neurodegeneration [206]. Aneuploidization of the brain in ataxia telangiectasia is a result of mutations in *ATM* (ataxia telangiectasia mutated) gene, which is involved in numerous molecular and cellular pathways (i.e., apoptosis, cell cycle, cellular senescence, cellular responses to stress, DNA damage response, DNA repair, etc.) [178,207,208,209]. Actually, CIN mediating neurodegeneration might be a drug target in ataxia telangiectasia [205,208]. In addition, since the main source for chromosomally instable genomes is the developing brain, in which cells with aneuploidy are suggested to be cleared [25,147], it has been proposed that uncleared brain cells affected by developmental aneuploidy/CIN are progressively dying later in life [180,181,206]. Likewise, developmental aneuploidization (if evolved clonally) without further prenatal clearance might be a candidate process for pediatric brain cancers, which are common in early childhood [210]. In this light, it would be important to know whether this mechanism is applicable to late-onset neurodegenerative diseases.

Alzheimer’s disease represents a picturesque example of late-onset neurodegenerative diseases associated with alterations to cell cycle regulation in the brain and genome instability [145,147,211,212]. It is important to mention that Alzheimer’s disease and Down syndrome (trisomy of chromosome 21) have certain similarities. Extra copy of chromosome 21 and, more importantly, *APP* gene with increased β-amyloid peptide production are probable mechanisms for the universal development of Alzheimer’s disease neuropathology and high risk of Alzheimer’s disease-like dementia in Down syndrome [211]. SCM (aneuploidy) has been shown to affect the Alzheimer’s disease brain [146,206]. Chromosome 21 specific mosaicism/instability has been found to be the commonest type of SCM/CIN in the Alzheimer’s disease brain [206]. Consequently, mosaic aneuploidy has been shown to initiate selective cell death, which is responsible for neurodegeneration in the diseased brain [181]. Mosaic X chromosome loss (a cytogenetic hallmark of aging [139]) has been revealed to be more common in the Alzheimer’s disease brain as to controls [213]. SCM/CIN seems to be area-specific and to correlate with neuronal vulnerability in the Alzheimer’s disease brain [214]. More specific types of instability (submicroscopic copy number and structure instability of the *APP* gene) have been discovered to affect the Alzheimer’s disease brain, as well [215,216]. Demonstrably, a study performed using single-cell whole genome sequencing have shown the lack of high rates of gross chromosomal imbalances in the Alzheimer’s disease brain in contrast to molecular neurocytogenetic studies [57,58,64]. To explain these differences, one can propose specificity of cohorts or technical limitations (e.g., whole-genome amplification, moderate cell scoring potential of single-cell whole genome sequencing). Although the reasons of the data discrepancies would be certainly revealed by forthcoming studies, we insist that the most valid results would be obtained by combining post-genomic and molecular cytogenetic (visualization) techniques.

Taking into account the knowledge about different pathways to Alzheimer’s disease neurodegeneration and genome instability, DNA replication stress hypothesis of Alzheimer’s disease has been proposed to explain the occurrence of SCM and CIN in post-mitotic cells of the diseased brain [215]. The hypothesis proposes cell cycle errors affecting DNA replication to produce the aforementioned types of SCM/CIN [144,217]. Alternatively, genome instability mediating Alzheimer’s disease neurodegeneration might result from abnormal centromere/cohesion dynamics producing cell cycle dysfunction and, thereby, aneuploidy [218,219]. Niemann-Pick C1 disease, Lewy body diseases, and frontotemporal lobar degeneration caused by *MAPT* mutations have also been associated with CIN/aneuploidy or mitotic defects and abnormal chromosome segregation resulting in neurodegeneration [220,221,222]. Molecular and cellular pathways to SCM/CIN-mediated neurodegeneration include not only mitotic/cell cycle errors but also abnormal programmed cell death, which is probably stimulated by mosaic aneuploidy [12,185,223]. In total, further post-genomic studies appear to be required for unraveling intrinsic neurodegenerative pathways and the role of SCM/CIN in devastative brain diseases.

Intercellular genome variations through the lifespan have been hypothesized to influence human behavior [7,224]. Morphological chromosome defects have been already shown to occur because of stress/extreme situations (i.e., individual suffering from gulf war illness) [225]. Addressing these ideas in the cyto(post)genomic context, a cytogenomic hypothesis has been proposed. Briefly, behavioral changes may correlate with dynamic variations of SCM/CIN rates throughout the lifespan. The hypothesis has been suggested to be useful for therapies to improve the overall condition of individuals with behavioral problems by decreasing mosaicism rates [226]. Taking into consideration the nature of genetic-environmental interactions affecting cellular genomes in brain diseases [185], the validity of this hypothesis is highly probable.

In the available literature, there are also reports on specific SCM and CIN in autoimmune disease [136,227,228], cutaneous disorders [229], and eye diseases [230]. Thus, studying tissue-specific SCM/CIN is able to unravel new molecular and cellular mechanisms of human diseases, as a whole. It is likely that further studies of genetic diseases would reveal additional pathologic conditions mediated by SCM and/or CIN.

It is important to stress that CIN and SCM are more likely to be an important element of pathogenetic cascade of cancer and brain diseases [15,149,159,171,185]. Therefore, the main phenotypic outcome in the aforementioned diseases is likely to be essentially determined by the changes at the chromosomal level caused by another genetic defect (i.e., specific mutations or “mutational burden”) or combination of genetic changes and genetic-environmental interactions [3,183,231,232]. Environmental influences are able to trigger a cascade of abnormal processes producing genome instability (i.e., brain-specific SCM/CIN) [185]. This idea underlies 2-hit/multihit hypothesis for complex diseases (cancer and mental illness) suggesting that CIN and SCM are secondary to genetic changes and/or genome interactions with environment [149,152,161,185]. Thus, similar types of SCM/CIN should not be observed in all the affected individuals/tissues. However, the overall contribution of all the SCM/CIN types to the etiology of complex and chromosomal diseases is likely to be substantial.

## 6. Technical Aspects of SCM/CIN Studies

As noted previously, technical aspects of SCM/CIN studies are extremely important and determine the value of data obtained [55,57,233]. The studies of SCM and CIN in numerous human tissues have long been performed exclusively by interphase cytogenetics, which is technologically synonymous to interphase FISH or FISH-based approaches [65,66,67,234]. To identify chromosomal variations in interphase by molecular cytogenetic methods, one can apply FISH-based methods (multiprobe interphase FISH) and chromosomal microarray techniques (i.e., array CGH or SNP/array) (for review, see [66,69,70,71,235]). Alternatively, single-cell sequencing is able to reveal the majority of genomic variations in non-dividing cells achieving the highest resolution level of cellular genome analysis [62,63,64,67]. However, a number of chromosomal variations or CIN (i.e., chromosomal fragility, heteromorphisms, and interphase chromosome breaks) cannot be uncovered by single-cell whole genome analysis [70]. To succeed in studying alterations to interphase chromosome morphology in large cell populations, interphase chromosome-specific multicolor banding or ICS-MCB, has been developed. This approach allows visualization of interphase chromosomes in their integrity at molecular resolution in single cells at any stage of the cell cycle [236,237,238]. It is based on the application of microdissection-engineered DNA probes and fluorescence multicolor chromosome banding (for more details, see [234]). A more sophisticated alternative to ICS-MCB may be the application of a wide panel of site-specific DNA probes for homologous chromosomes [239]. Recently, we have thoroughly reviewed FISH-based methodology for studying SCM/CIN in humans [57,240,241].

An important technical issue for any molecular cytogenetic study dedicated to the analysis of large cell populations acquired from different tissues is the preparation of cellular suspensions [57,58,242]. For instance, brain tissue preparations for molecular neurocytogenetic analysis has its own specificity [243,244]. Finally, chromosomal arrangement in post-mitotic cells may mimic chromosome imbalances and/or CIN (i.e., somatic pairing of chromosomal regions and chromosome loss) [245]. To solve the problem, quantitative FISH, which allows for a discrimination between chromosomal associations and chromosome loss, may be applied [246,247]. Quantitative FISH with PNA (peptide nucleic acid) probes is a highly effective approach to a common CIN type referred to as telomere length variations (shortening) [234,242]. It is to note that molecular cytogenetic methodology for visualizing interphase chromosomes cannot be completely substituted by single-cell whole genome scanning techniques [241]. Although post-genomic technologies allow single-cell genomic analysis at the highest resolution possible, uncovering chromosomal intercellular heterogeneity still requires visualization of chromosomal loci by cytogenetic karyotyping and FISH-based methods [234,248,249].

Recently, we have proposed a workflow for studies of mosaic aneuploidy and chromosome instability for uncovering molecular/cellular disease mechanisms in the post-genomic era (i.e., cytopostgenomic studies). The term cytopostgenomics have been coined to cover an emerging bioscience field dedicated to postgenomic analysis focused on causes and consequences of chromosomal variations and architecture [248]. According these considerations, high-resolution whole genome scanning technologies and post-genomic bioinformatic approaches (specifically developed for molecular cytogenetics, i.e., [149,165,167,180]) are to be used to reveal molecular causes of chromosomal heterogeneity identified by cytogenetic and molecular cytogenetic techniques [241,248,249]. In total, it seems that a successful study of SCM/CIN is likely to be a result of a comprehensive combination of cytogenetic, molecular cytogenetic and post-genomic technologies.

## 7. Chromosomal Mosaicism and Aging

SCM is the commonest type of ontogenetic genome variations associated with aging [10,16,250]. Cells affected by gonosomal monosomy have long been found to accumulate with aging; more precisely, the accumulation of cells exhibiting X chromosome loss has been observed to progress with age [139,251]. Alzheimer’s disease—a disease associated with aging—seems to be exhibit an increase in the rates of mosaic X chromosome loss [213,217,252]. Furthermore, there are numerous observations that shared mechanisms underlie SCM/CIN in cancer, neurodegeneration and aging [10,16,253,254]. In mitotic cells, aging-dependent accumulation of chromosomal mutations is the result of abnormal chromosome segregation during cell division, which might result from exhausting of mitotic checkpoint machinery. However, similar processes seem to occur in populations of post-mitotic cells (i.e., in the brain), as well [253,254,255]. The hypothesis for explaining this paradox has suggested CIN (genomic instability) generated during erroneous re-enter to the cell cycle, which might result in accumulation of neural aneuploidy with aging [255]. Interestingly, there are several reports showing increased rates of low-level mosaic aneuploidy and DNA content variations in the aging brain [256,257,258]. CIN and telomere lengths variations are shown to hallmark aging and cellular senescence [259]. Therefore, it appears that SCM and CIN may be related to tissue and cell senescence producing aging phenotypes.

**Figure 3:**
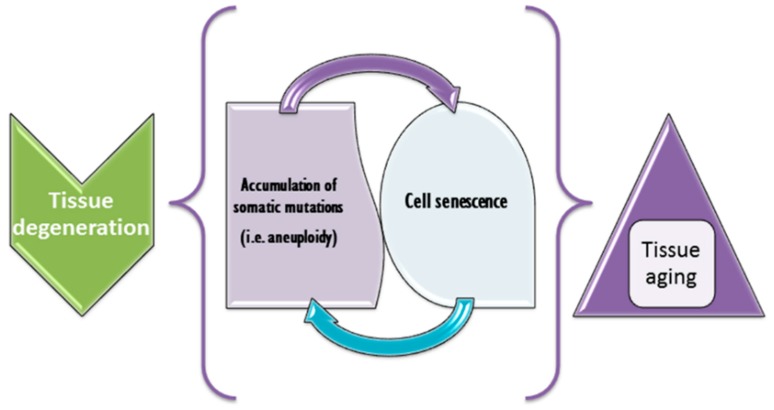
Hypothetical role of SCM/CIN in aging. It appears that accumulation of somatic mutations (e.g., aneuploidy, alterations to chromosome structure, repeat length variations, intragenic sequence changes, etc.) interplays with cell senescence. The result of this interplay might produce either aging at the tissular level or tissue degeneration, which is likely to be responsible for aging of an organism, as a whole.

Cell senescence has been repeatedly shown to hallmark cell populations affected by CIN (mainly aneuploidy) [260,261,262]. The co-occurrence of cell senescence and CIN/aneuploidy is generally used to explain progeroid phenotypes in aneuploidy and CIN syndromes [16,253]. On the other hand, aging is supposed to be associated with progressive accumulation of somatic mutations [263]. Microscopic and submicroscopic chromosomal variations seem to represent the commonest types of such mutations in the human brain [256,264]. However, single cell sequence analysis has demonstrated the rarity of complex karyotypic changes in aged human neocortex [265]. Nonetheless, SCM/CIN/aneuploidy (accumulation of somatic chromosomal mutations) and cell senescence are likely to be tightly interconnected to each other. Schematically, this interconnection is shown in Figure 3. The nature of the interconnection is certainly important for understanding aging at cellular and tissular levels. To this end, it is again to mention that combining molecular cytogenetic and post-genomic methodologies is the most promising way to evaluate causes and consequences of SCM and CIN. Such future studies are able to determine the underlying genomic causes of cell senescence and aging in mitotic and post-mitotic human cell populations.

## 8. Conclusions

Chromosomal heterogeneity mediated by somatic mosaicism and CIN appears to be relevant to a variety of biological processes and medical conditions. Regardless of numerous studies of genome instability manifesting as SCM, the intrinsic incidence remains to be established. CIN has been mainly evaluated in early embryos, cancer, and a number of brain diseases. Thus, the presence of CIN in unaffected human tissues and its possible contribution to non-cancerous diseases is almost unknown.

CIN and SCM appear to be involved in early prenatal development. In addition, developmental CIN might be a mechanism for natural selection and diseases. It is highly probable that the mechanism is realized by a bottleneck effect. Furthermore, the rates of SCM or CIN are ontogenetically variable. These somatic genome variations seem to mediate aging processes at cellular and tissular levels.

SCM and CIN are likely to be elements of a global pathogenetic cascade in complex diseases (e.g., cancer and mental illness). To investigate the origins and consequences of SCM/CIN, post-genomic approaches (whole-genome and system biology analyses) are certainly required. Thus, individuals and cell populations exhibiting SCM and/or CIN are to be scanned using post-genomic techniques to reveal non-mosaic genomic variations, which may render susceptible cellular genomes to the instability. Additionally, future studies of SCM and CIN may uncover how similar mechanisms involving SCM/CIN lead to different clinical conditions (i.e., neurodevelopmental diseases, neurodegenerative disorders, or cancer). Finally, it is important to add that post-genomic techniques (e.g., single-cell next generations sequencing) are unable to completely substitute for visualization molecular cytogenetic techniques (i.e., banding karyotyping and FISH-based methods), inasmuch as several types of CIN are undetectable by sequencing and microarray technologies. To this end, we expect that further evaluations of SCM/CIN in health and disease open bright perspectives for genomic research.
